# Supplementary material for: Synergistic Effect of Motivation for the Elderly and Support for Going out
Source: J Pers Med. 2022 Jul 30;12(8):1257. doi: 10.3390/jpm12081257 (PMC9410321; doi:10.3390/jpm12081257)
Supplement: Supplementary file 1 [file jpm-12-01257-s001.zip › jpm-1836601-supplementary/Supplement figure S1.pdf]

Distribution of FIM total scores

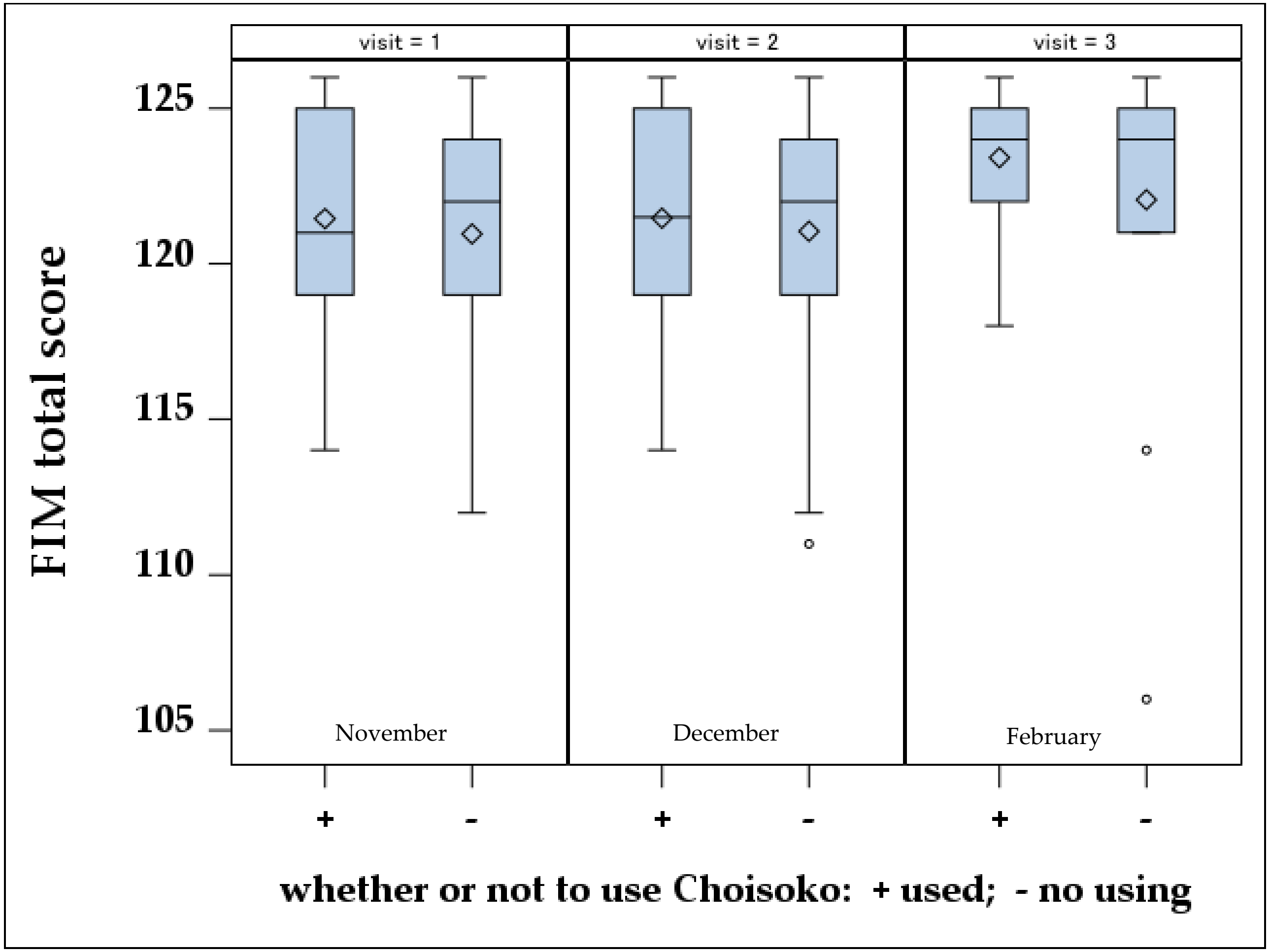

Supplement figure S1: Among the subjects who used the Choisoko system, those with low FIM scores in December showed an improvement in FIM scores in February.
