# Supplementary material for: Synergistic Effect of Motivation for the Elderly and Support for Going out
Source: J Pers Med. 2022 Jul 30;12(8):1257. doi: 10.3390/jpm12081257 (PMC9410321; doi:10.3390/jpm12081257)
Supplement: Supplementary file 1 [file jpm-12-01257-s001.zip › jpm-1836601-supplementary/Supplement table S1.pdf]

Supplement table S1 Comparisons of changes in measurements at the first two points

	Choisoko use (N=31)		Non Choisoko use (N=25)		
	N		N		P-value
Grip strength (right)	30	-0.67 ± 2.08	22	0.14 ± 2.67	0.243
Grip strength (left)	30	0.11 ± 2.82	22	0.79 ± 1.99	0.314
Grip strength max	30	-0.32 ± 2.16	22	-0.11 ± 2.08	0.734
Stand up	30	-2.01 ± 2.55	20	-1.98 ± 2.85	0.967
Walk time	30	0.06 ± 0.94	21	-0.38 ± 1.13	0.159
Walk speed	30	-0.01 ± 0.17	21	0.04 ± 0.16	0.332
Walk time max	30	0.02 ± 0.42	21	-0.33 ± 0.59	0.026
Walk speed max	30	-0.02 ± 0.14	21	0.07 ± 0.12	0.019
Weight	28	0.39 ± 0.66	21	0.62 ± 1.95	0.603
BMI	28	0.17 ± 0.30	21	0.30 ± 0.93	0.566
Body fat	28	-0.64 ± 1.54	21	-0.14 ± 2.14	0.374
Muscle	28	0.55 ± 0.74	21	0.28 ± 0.78	0.221
Quadmus	28	0.13 ± 0.29	22	0.13 ± 0.18	0.999
Alb	30	0.02 ± 0.18	20	0.10 ± 0.20	0.207
GOT	30	-1.37 ± 4.70	20	0.65 ± 3.01	0.071
GPT	30	-2.00 ± 5.30	20	0.00 ± 2.34	0.076
BUN	30	0.54 ± 3.31	20	3.18 ± 6.45	0.103
CRE	30	0.01 ± 0.07	20	0.00 ± 0.05	0.719
eGFR	30	-0.18 ± 6.63	20	-0.35 ± 4.05	0.913
WBC	30	450.00 ± 885.81	20	155.00 ± 591.59	0.164
RBC	30	-12.98 ± 71.10	20	26.12 ± 90.01	0.112
Hb	30	0.07 ± 0.46	20	0.17 ± 0.50	0.465
Ht	30	0.06 ± 1.48	20	0.66 ± 1.48	0.166
MCV	30	0.25 ± 1.71	20	0.39 ± 1.34	0.762
MCH	30	0.20 ± 0.65	20	0.01 ± 0.45	0.216
MCHC	30	0.13 ± 0.70	20	-0.10 ± 0.49	0.175
Platelet	30	0.35 ± 1.65	20	0.50 ± 2.49	0.820
Total of basic check	30	-2.27 ± 2.85	21	-1.10 ± 1.30	0.055
Volume of iliopsoas muscle	28	-0.06 ± 2.11	23	0.25 ± 2.93	0.673

Data are presented as mean ± standard deviation

Alb: albumin, AST: aspartate aminotransferase, ALT: alanine aminotransferase, BUN: blood urea

nitrogen, CRE: creatinine, eGFR: estimated glomerular filtration rate, WBC: White blood cell, RBC: Red blood cell, Hb: hemoglobin, Ht: hematocrit, MCV: mean corpuscular volume, MCH: mean corpuscular hemoglobin, MCHC: mean corpuscular hemoglobin concentration.
