# Supplementary material for: Synergistic Effect of Motivation for the Elderly and Support for Going out
Source: J Pers Med. 2022 Jul 30;12(8):1257. doi: 10.3390/jpm12081257 (PMC9410321; doi:10.3390/jpm12081257)
Supplement: Supplementary file 1 [file jpm-12-01257-s001.zip › jpm-1836601-supplementary/Supplement table S2.pdf]

Supplement table S2 Comparisons of changes in measurements at the last two points

	Choisoko use (N=31)		Non Choisoko use (N=25)		
	N		N		P-value
Grip strength (right)	28	-0.41 ± 2.36	17	-1.16 ± 2.51	0.329
Grip strength (left)	28	-0.93 ± 1.50	17	-1.39 ± 1.72	0.361
Grip strength max	28	-0.78 ± 1.94	17	-1.04 ± 2.33	0.702
Stand up	28	-0.84 ± 1.50	15	-1.34 ± 1.94	0.391
Walk time	28	-0.48 ± 0.90	16	-0.04 ± 1.45	0.287
Walk speed	28	0.06 ± 0.12	16	0.07 ± 0.12	0.891
Walk time max	28	-0.42 ± 0.43	16	-0.24 ± 0.30	0.101
Walk speed max	28	0.12 ± 0.13	16	0.08 ± 0.10	0.244
Weight	27	-0.49 ± 2.04	17	0.27 ± 0.88	0.100
BMI	27	-0.25 ± 1.01	17	0.12 ± 0.40	0.099
Body fat	27	-0.62 ± 2.78	17	0.92 ± 2.06	0.042
Muscle	27	-0.10 ± 0.89	17	-0.26 ± 0.61	0.472
Quadmus	26	0.00 ± 0.32	16	-0.09 ± 0.18	0.266
Alb	28	-0.03 ± 0.21	13	-0.05 ± 0.17	0.638
GOT	28	0.68 ± 4.46	13	0.00 ± 4.26	0.644
GPT	28	-0.86 ± 5.90	13	-0.77 ± 3.61	0.954
BUN	28	1.66 ± 3.19	13	-2.52 ± 7.73	0.081
CRE	28	-0.02 ± 0.08	13	-0.04 ± 0.04	0.185
eGFR	28	2.29 ± 7.14	13	3.32 ± 3.22	0.528
WBC	28	-182.14 ± 910.86	13	246.15 ± 638.51	0.092
RBC	28	16.27 ± 79.15	13	-2.15 ± 13.09	0.241
Hb	28	-0.09 ± 0.54	13	-0.27 ± 0.40	0.236
Ht	28	0.26 ± 1.93	13	-0.27 ± 1.53	0.354
MCV	28	-0.03 ± 2.10	13	-0.06 ± 1.47	0.954
MCH	28	-0.43 ± 0.78	13	-0.42 ± 0.61	0.993
MCHC	28	-0.44 ± 0.82	13	-0.48 ± 0.67	0.889
Platelet	28	-0.01 ± 1.89	13	0.41 ± 1.90	0.514
Total of basic check	28	-1.57 ± 2.59	17	-1.47 ± 2.43	0.896
Volume of iliopsoas muscle	26	0.89 ± 3.14	19	0.52 ± 3.10	0.697

Data are presented as mean ±standard deviation

Alb: albumin, AST: aspartate aminotransferase, ALT: alanine aminotransferase, BUN: blood urea

nitrogen, CRE: creatinine, eGFR: estimated glomerular filtration rate, WBC: White blood cell, RBC: Red blood cell, Hb: hemoglobin, Ht: hematocrit, MCV: mean corpuscular volume, MCH: mean corpuscular hemoglobin, MCHC: mean corpuscular hemoglobin concentration.
