# Supplementary material for: Synergistic Effect of Motivation for the Elderly and Support for Going out
Source: J Pers Med. 2022 Jul 30;12(8):1257. doi: 10.3390/jpm12081257 (PMC9410321; doi:10.3390/jpm12081257)
Supplement: Supplementary file 1 [file jpm-12-01257-s001.zip › jpm-1836601-supplementary/Supplement Table S3.pdf]

Supplement Table S3 Comparisons of FIM score changes at the first two points

	Choisoko use (N=31)		Non Choisoko use (N=25)			
Change of FIM score	N	Mean ±SD	N	Mean ±SD	Difference (95%CI)	P-value
Total score	30	“U+2212”0.03±0.18	22	“U+2212”0.05±0.49	0.01 (“U+2212”0.21 to 0.24)	0.912
Exercise score	30	“U+2212”0.07±0.37	22	“U+2212”0.09±0.43	0.02 (“U+2212”0.20 to 0.25)	0.831
Cognitive score	30	0.03±0.41	22	0.05±0.21	“U+2212”0.01 (“U+2212”0.19 to 0.17)	0.891

SD: Standard deviation CI: Confidence Interval
